# Assessment of genetic structure and trait associations of Watkins wheat landraces under Egyptian field conditions

**DOI:** 10.3389/fgene.2024.1384220

**Published:** 2024-12-02

**Authors:** Ahmed Fawzy Elkot, Ahmed E. Nassar, Elsayed L. Elmassry, Macarena Forner-Martínez, Rajani Awal, Luzie U. Wingen, Simon Griffiths, Alsamman M. Alsamman, Zakaria Kehel

**Affiliations:** ^1^ Wheat Research Department, Field Crops Research Institute, Agricultural Research Center, Giza, Egypt; ^2^ Agricultural Genetic Engineering Research Institute (AGERI), Agricultural Research Center (ARC), Giza, Egypt; ^3^ International Center for Agricultural Research in the Dry Areas (ICARDA), Rabat, Morocco; ^4^ John Innes Centre, Norwich Research Park, Norwich, United Kingdom

**Keywords:** wheat, genome-wide association study (GWAS), Watkins, marker trait associations, population structure, agromorphological traits

## Abstract

**Background:**

Wheat landraces represent a reservoir of genetic diversity that can support wheat improvement through breeding. A core panel of 300 Watkins wheat landraces, as well as 16 non-Watkins landraces and elite wheat cultivars, was grown during the 2020–2021 and 2021–2022 seasons at four Agricultural Research Stations in Egypt, Gemmiza, Nubaria, Sakha, and Sids, to evaluate the core panel for agromorphological and yield-related traits. The genetic population structure within these genotypes were assessed using 35,143 single nucleotide polymorphisms (SNPs).

**Results:**

Cluster analyses using Discriminant Analysis of Principal Components (DAPC) and k-means revealed three clusters with moderate genetic differentiation and population structure, possibly due to wheat breeding systems and geographical isolation. The best ancestry was k = 4, but k = 2 and k = 3 were also significant. A genome-wide association study (GWAS) identified clustered marker trait associations (MTAs) linked to thousand kernel weight on chromosome 5A, plant height on chromosomes 3B and 1D, days to heading on chromosomes 2A, 4B, 5B and 1D, and plant maturity on chromosomes 3A, 2B, and 6B. In the future, these MTAs can be used to accelerate the incorporation of beneficial alleles into locally adapted germplasm through marker-assisted selection. Gene enrichment analysis identified key genes within these loci, including Reduced height-1 (Rht-A1) and stress-related genes.

**Conclusion:**

These findings underscore significant genetic connections and the involvement of crucial biological pathways.

## 1 Introduction

Wheat is Egypt’s main food crop and one of the oldest cultivated cereal crops, with evidence of its use for bread making dating back to 7300-6000 B.P (Boulos and Fahmy, 2007). Wheat accounts for 40% of the protein and 37% of the calories in the Egyptian diet (Abdalla et al., 2023). More than 70 million Egyptians rely solely on bread, consuming five loaves per person each day (Abay, 2023). The wheat-growing area covers 1.5 million hectares – 33% of the cultivated area during the winter season–and yielded approximately 10 million tons in 2022. Notwithstanding, to sustain national demand, Egypt imports 12.5 million tons of wheat annually, making it the world’s largest importer of wheat (Aalto et al., 2022). Despite the government’s food security policy, which has led to a production growth rate of 4.1% per year, leading to an overall 4.3-fold grain yield increase since 1981, there is an urgent need to further increase wheat production (Abdelmageed et al., 2019).

Climate change (Filho, 2015), limited water resources (Yigezu et al., 2021), and limited agricultural growth present challenges and threats to wheat production in Egypt. About 85% of water consumption in Egypt is used in agriculture, while the remaining is allocated to urban areas (Gabr et al., 2024). Climate change is widely accepted as the most pressing environmental issue concerning the entire planet (El Massah and Omran, 2015). Through temperature increases, altered precipitation patterns, elevated CO_2_ levels, high evaporation rates, increased pest and disease prevalence, and other consequences (Filho, 2015), it is estimated that by 2050, cereal crop production will decline by 18% for wheat and barley, 19% for maize and sorghum, 28% for soybeans, and 11% for rice (Wang et al., 2018). To address the challenges facing wheat production in Egypt and mitigate against the impact of climate change, the national wheat breeding program is focused on releasing new wheat varieties with improved disease resistance, a more desirable root architecture, heavier grains, resistance to more extreme temperatures, and lower water requirements.

Wheat researchers and breeders make use of various genotyping techniques, such as single-nucleotide polymorphism (SNP) hybridization arrays, to link genotypic variants to phenotypic traits to improve crop yields, increase agricultural diversity, and support more sustainable farming practices. SNPs are the most commonly used molecular markers due to their wide availability across all genomes and low cost compared to other marker technologies (Broccanello et al., 2018). They can be used to create arrays of thousands of markers spread across the whole genome, even for very large autopolyploid or allopolyploid species (Ganal et al., 2012; Allen et al., 2017). For wheat, the benefits of understanding and making full use of genetic diversity are immense, primarily in terms of increasing production. By studying the interactions between genotypes and their surroundings, wheat can be bred for improved pathogen resistance, climate change adaptation, and superior production characteristics. The use of SNP markers for association and linkage mapping studies has greatly enhanced the research potential of wheat (Lucas et al., 2017). SNP markers, wheat functional and comparative genomics, and marker-assisted selection (MAS) are all tools that can be used to help overcome the limitations of traditional breeding methods, by streamlining the integration of genes to improve disease resistance, increase yield, and improve quality (Rasheed et al., 2018).

Here, we explored the genetic structure, relationship, and potential importance of 300 core Watkins wheat landraces that were previously selected to maximize genetic information (Arora et al., 2023; Wingen et al., 2014). The panel was supplemented with 16 non-Watkins landraces and elite cultivars. Comprehensive genetic structure, k-means cluster, and population structure analyses were conducted to understand the genetic content in the panel. Additionally, we performed genome-wide association studies (GWAS) with 35,143 SNP markers and identified marker trait associations (MTAs) with date of heading, date of maturity, plant height, and thousand kernel weight. Our study contributes to the understanding of how wheat genetics interacts with the environment to affect wheat cultivation in Egypt. The results of this study can be used to inform the development of new wheat varieties better adapted to Egyptian cultivation conditions.

## 2 Materials and methods

### 2.1 Plant materials, field site locations, and phenotypic data assessment

**FIGURE 1:**
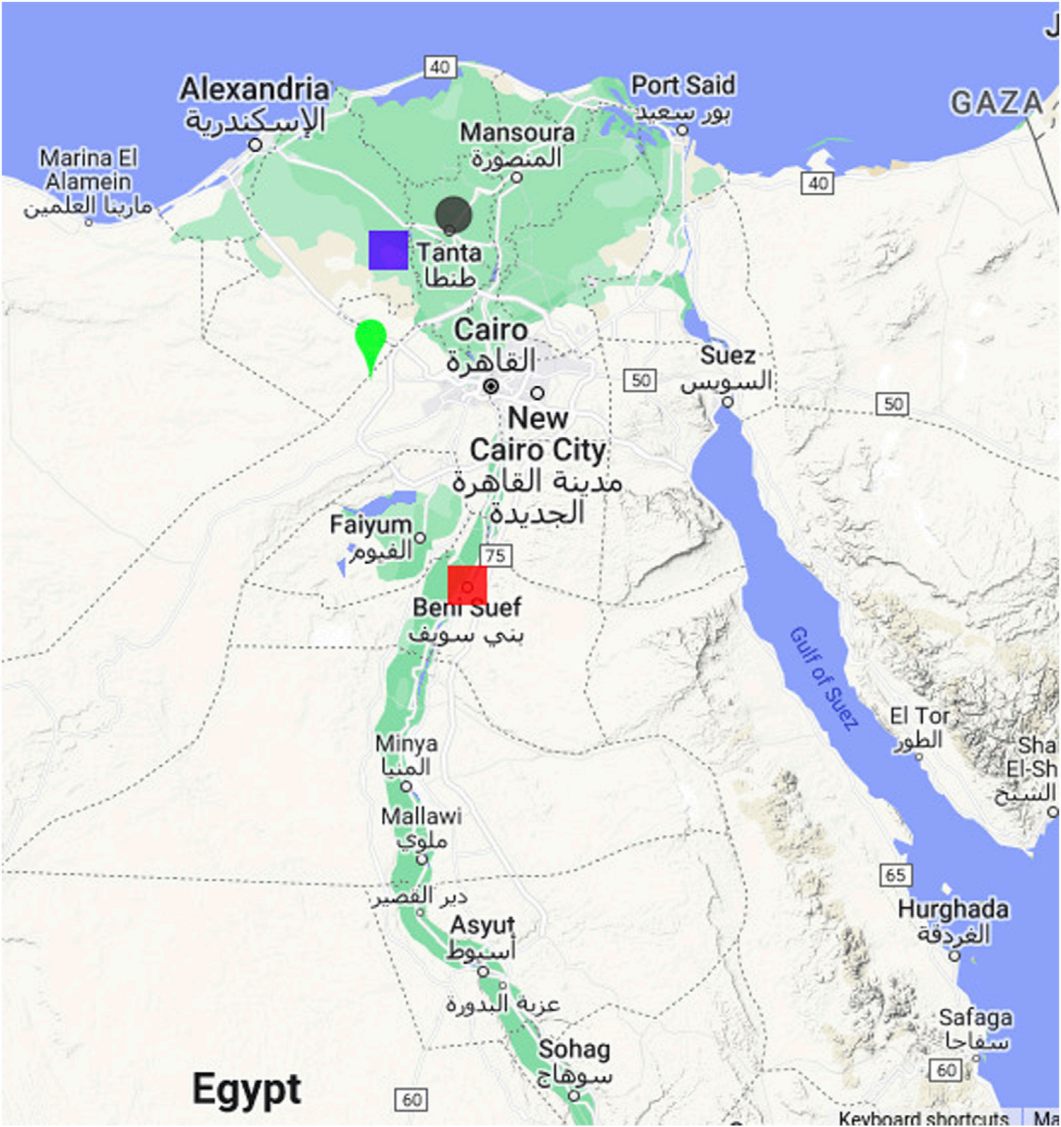
Map showing the locations of the experimental field trials conducted for wheat in Egypt. The locations include the Sakha Agricultural Research Station (green teardrop), Nubaria Agricultural Research (mauve square), Gemmiza Agricultural Research Station (grey spot), and Sids Agricultural Research (red square).

**FIGURE 2:**
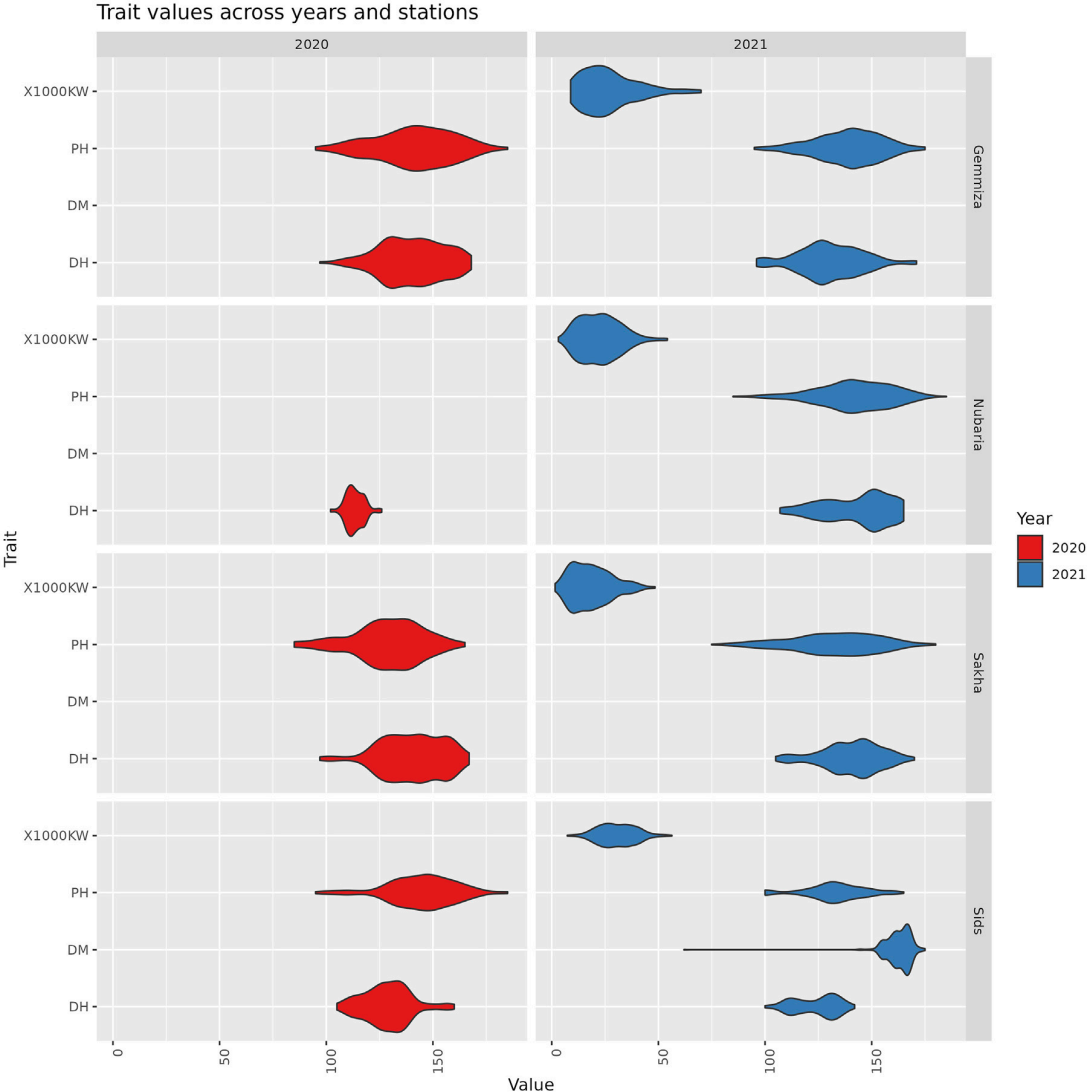
The distribution of trait values across different years and stations. Each violin represents the range and density of trait values for a specific combination of trait, station, and year.

The plant materials used in the current study comprised 300 Watkins wheat landraces and 16 non-Watkins landraces and elite cultivars obtained from the Germplasm Resource Unit (GRU) at the John Innes Centre in Norwich, United Kingdom (Supplementary Table S1). The 316 lines were grown in two consecutive seasons, namely 2020–2021 and 2021–2022, in four different agricultural research stations in Egypt with varying environmental conditions, including 1) Sakha Agricultural Research Station in the north Delta (30.0642 ° N, 30.5645 ° E), 2) Nubaria Agricultural Research Station in the West Nile Delta to represent newly reclaimed lands under surface irrigation (30.6973 ° N, 30.66713 ° E), 3) Gemmiza Agricultural Research Station in the Middle Delta to represent heavy and fertile soil (30.867 ° N, 31.028 ° E), and 4) Sids Agricultural Research Station in Middle Egypt, a region characterized by high yielding conditions in the Nile valley (29.076 ° N, 31.097 ° E) (Figure 1). Phenotypic data were collected as the number of days to heading, plant height, plant growth habit, and thousand kernel weight (1,000 KW). The phenotypes were obtained by growing each entry in 3-metre-long rows 30 cm apart. Fertilizers were applied by adding phosphorus fertilizer at 35 kgP ha^−1^ one dose before sowing, and nitrogenous fertilizer at 180 kgN ha^−1^ in three separate doses during sowing, 30 days post-sowing, and at the tillering stage. The sowing date was 15 November every season. The agronomic characteristics were recorded as days to heading (DH), the days to heading was recorded as heading when the spike emerged for 0.25 of its length in 50% of the plants (Zadoks DGS 53) and, plant height in cm (PH), was measured as PH, from the soil surface to spike top, and thousand kernel weight (1,000 KW; g) (Figure 2).

### 2.2 Statistical analysis

The screening traits were consecutive years to examine the phenotypic variation across four diverse ranges of growing conditions under natural field condition in Egypt in conductive 2 years. The mean was calculated as the average of ten plants in each row. The graphical analysis of the evaluated genotypes, including genotype-by-environment (GGE) biplots for days to heading (DH), plant height (PH), and thousand kernel weight (1,000 KW) across four environments over two consecutive seasons, was conducted using GenStat 19th Edition (VSN International Ltd., Hemel Hempstead, United Kingdom) following the methodology of Yan et al. (2000), Yan et al. (2007). The sowing date was 15 November every season. The agronomic characteristics were recorded as days to heading (DH), the days to heading was recorded as heading when the spike emerged for 0.25 of its length in 50% of the plants (Zadoks DGS 53) and, plant height in cm (PH), was measured as PH, from the soil surface to spike top, and thousand kernel weight (1,000 KW; g). A correlation matrix was calculated to determine the degree of correlation between the location and the phenotypes. The two stability parameters of superiority performance (Lin and Binns, 1988) and mean ranks (Nassar and Huehn, 1987) were used to quantify and rank wheat genotypes for good performance and stability where a genotype had the lowest values of the two parameters is considered the most stable one.

### 2.3 Genotyping

We downloaded the Watkins SNP genotype data from the Cereals Data Base (https://www.cerealsdb.uk.net/cerealgenomics/cgi-bin/display_varieties.pl?example=35K_breeders_array&submitter=Submit+Button). This data had previously been generated using the Affymetrix 35K Axiom® Wheat Genotyping Array and screened for genetic variations across the A, B, and D genomes (Allen et al., 2017). TASSEL software (version 5) (Bradbury et al., 2007) was employed to remove SNPs with a minor allele frequency (MAF) lower than 0.05 and a call rate below 90%.

### 2.4 Population structure analyses

The genetic structure of bread wheat was evaluated using samples from 33 diverse nations in Europe, Asia, and Africa. The samples included 56 landraces from India, 32 from China, 28 from Spain, and others (Supplementary Table S1). Discriminant analysis of principal components (DAPC) was used to identify the differences among populations and generate summarized features for cluster analysis. It was performed without prior information on individual populations using Adegenet v2.1.3 (Jombart, 2008). The find.clusters function was utilized to confirm the DAPC results and determine the optimal number of clusters (k) using three different plots. The first plot was based on the lowest value in the “elbow” of the Bayesian information criterion (BIC) curve, the second on a scatter plot of the discriminant functions (DAPC), and the third on a bar plot of each sample’s posterior probabilities of group assignment. To determine the genetic distance between countries, the dist function in the Stats v4.0.3 library (Pellegrini and Dusanter-Fourt, 1997) was utilized. The hclust (Hierarchical Clustering) function was used to determine cluster distance and sample linkage, and the As.phylo function in the ape v5.4.1 library (Paradis et al., 2004) reformat the dendrogram into tree format. The annotated dendrogram was plotted using ITOL software (Letunic and Bork, 2019).

The population structure analysis was employed as a covariate in the GWAS analysis. It was conducted to assess genetic admixture, which refers to the process or outcome of interbreeding between multiple isolated populations within a species (Yuan et al., 2017). The LEA R package version 3.2.0 was used to examine the genetic structure of the core collection. The Snmf (sparse nonnegative matrix factorization) function was utilized within the package, with the number of ancestral genetic groups (K) varied from 1 to 10 in ten repeat runs for each K value. The function provided least-squares estimates of ancestry proportions instead of maximum likelihood estimates (François and Durand, 2010). The cross-entropy function was utilized to determine the optimal number of ancestral populations that best explain the genotypic data. The results were presented in a bar plot showing the Q-matrix constructed by the Q() function.

### 2.5 Genome-wide association and gene ontology analyses

In the current investigation, a GWAS was conducted using the Python-based tool vcf2gwas (Vogt et al., 2022). This tool implements the genome-wide efficient mixed model association (GEMMA) protocol and performs GWAS directly from a VCF file, while also allowing for multiple post-analysis operations (Zhou and Stephens, 2012). The GWAS analysis was executed using a linear mixed model (LMM) and the significant marker-trait associations were determined using the Wald test. The relevance of SNP-trait associations was determined based on the significance threshold of p -value ≤0.001 and False Discovery Rate (FDR) ≤0.1 displayed on the Manhattan plot. The results were visualized by generating Manhattan plots using the qqman and ggplot2 R packages. The TASSEL program (Bradbury et al., 2007) was used to calculate linkage disequilibrium (LD).

## 3 Results

### 3.1 Collection of phenotypic traits and their correlation between field sites and seasons

To study the phenotypic variation in the panel under Egyptian field conditions, the panel was grown for two seasons in four agricultural research stations in Sakha, Gemmiza, Sids, and Nubaria. We evaluated the panel for days to heading, days to maturity, plant height, and thousand kernel weight. We classified the correlations between the traits and locations, considering only values greater than 0.5 as significant due to the large sample size of the dataset. In the first season, a highly significant and positive correlation (r>0.6) was consistently observed for the number of days to heading across the four locations, indicating a similarity in climate during the heading stage across these regions. This positive and highly significant correlation persisted in the second season, particularly in Nubaria when compared to the other regions (Table 1). Regarding plant height, significant and positive correlations were found between Gemmiza and Sakha in the first season, and between Gemmiza and Nubaria in the second season (Table 2). However, for the 1,000 kernel weight trait, despite its statistical significance, the associations among this trait in the four locations were weak (r<0.5) (Table 3). This suggests that the trait is influenced by environmental factors that may differ between the four locations, such as temperature variation during the grain filling period and the severity of diseases. In summary, a significant correlation was generally observed between the traits as well as across the four field trial locations, except for thousand kernel weight (Table 3).

**TABLE 1:** Correlation matrix of the days to heading trait between the four field trial locations during the growth seasons 2020–2021 and 2021–2022.

Locations	Gemmiza	Nubaria	Sakha	Sids
Gemmiza	1	0.639**	0.739**	0.687**
Nubaria	0.256**	1	0.617**	0.664**
Sakha	0.825**	0.248**	1	0.640**
Sids	0.784**	0.239**	0.763**	1

** means significant relationships.

**TABLE 2:** Correlation matrix among plant height between the four field trial locations during the 2020–2021 (upper diagonal) and 2021–2022 (below diagonal) growing seasons.

Locations	Sakha	Gemmiza	Sids	Nubaria
Sakha	1	0.522**	0.378**	—
Gemmiza	0.444**	1	0.467**	—
Sids	0.206**	0.295**	1	—
Nubaria	0.476**	0.508**	0.316**	1

** means significant relationships.

**TABLE 3:** Correlation matrix of the 1,000 kernel weight trait between the four field trial locations during the 2021–2022 growing season.

Locations	Sakha	Sids	Nubaria	Gemmiza
Sakha	1			
Sids	0.293**	1		
Nubaria	0.478**	0.450**	1	
Gemmiza	0.018 ns	0.014 ns	−0.006 ns	1

Asterisks denote significant relationships between farmed landraces, whereas (ns) denotes a non-significant correlation among plants in the referred stations.

### 3.2 Stability parameters and genotype × environment (G × E) biplot

The two stability parameters of superiority performance [20] and mean ranks [21] were used to quantify and rank wheat genotypes for good performance and stability where a genotype had the lowest values of the two parameters is considered the most stable one (Lin and Binns, 1988; Nassar and Huehn, 1987). Genotype stability itself is not enough as a selection parameter for the aimed genotype unless its performance is good. Bearing in mind that the shortest and earliest genotypes plus that had the heaviest 1,000 kernel weight were desirable. In addition, the stability phenomenon was diagrammatically plotted using Genotype x Environment (G × E) biplot graph. In the case of heading date and plant height, as mentioned earlier the elite genotype must record the lowest values of these traits (below-grand mean that is located on the left side of the origin point in the GGE biplot graph) while the elite genotype regarding 1,000 kernel weight that surpassed the grand mean and laid out on the right side of the GGE biplot graph. Estimates of superiority performance and mean ranks of 30 selected wheat genotypes for heading date, plant height and 1,000 kernel weight are summarized in (Supplementary Tables S2–S4). Results shown in Supplementary Table S1 - Supplementary Figure S7 indicated 30 wheat genotypes that recorded the earliest heading date (less than the grand mean 132.5 days) as well as they recorded the lowest values of the two stability parameters being superiority performance and mean ranks. It is noted that 27 out of 30 wheat genotypes were stable using both stability parameters plus GxE biplot graph. Because the number of genotypes is more than 300, it is difficult to read the results from the crowded biplot graph.

Regarding plant height, there were 26 out of 30 wheat genotypes were stable using both numerical stability parameters and graphical method of GxE biplot graph (Table 1; Supplementary Figure S8). For 1,000 kernel weight, 25 genotypes were characterized by stability using the three methods of stability (Table 2; Supplementary Figure S9). Nine genotypes were revealed stability toward all tested agronomic characters (PH, DH and 1,000 KW) across location, namely, BecardKachu, CIMCOG_3, CIMCOG_32, CIMCOG_47, CIMCOG_49, CIMCOG_53, Reedling, Super 152 and Waxwing. While six wheat genotypes BAJ, CIMCOG_56, MISR1, Pfau, Weebill and Wyalkatchem were revealed stability toward plant height and days to heading in all different locations.

### 3.3 Characterization of genetic variation and linkage disequilibrium

**FIGURE 3:**
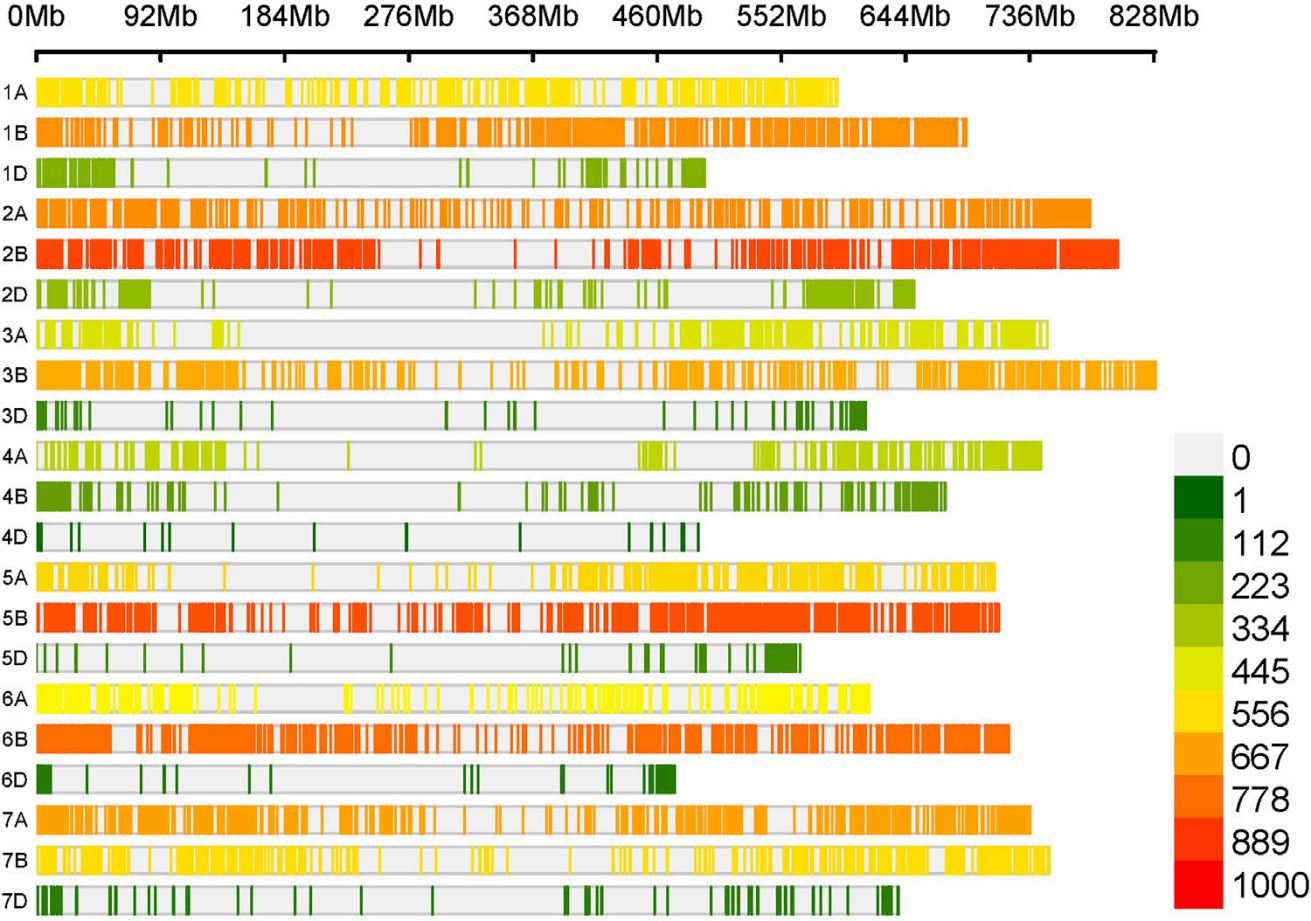
The distribution of 9402 SNP markers across the wheat genome. The x-axis represents the chromosomes of the wheat genome, while the vertical bars represent the density of SNP markers per Gb according to the legend on the right.

The Watkins wheat landrace collection is composed of 826 worldwide wheat genotypes collected during the 1930s before the onset of intensive breeding (Wingen et al., 2014). In the present study, we used a core set of 300 Watkins accessions previously selected to maximize the genetic diversity of the collection (Arora et al., 2023) and supplemented this with 16 additional entries (Supplementary Table S1). To elucidate the genetic diversity, structure, and relatedness of these 316 genotypes, we analyzed publicly available genotype data previously obtained with the Affymetrix 35K Axiom Wheat Breeder’s Genotyping Array. We filtered this data to retain 9402 SNP markers with a minor allele frequency (MAF) of less than 0.01 and a genotyping call rate of at least 90%. The number of SNP markers within 1,000 Mbp ranged from 36 (chromosome 4D) to 855 (chromosome 2B) (Figure 3).

**FIGURE 4:**
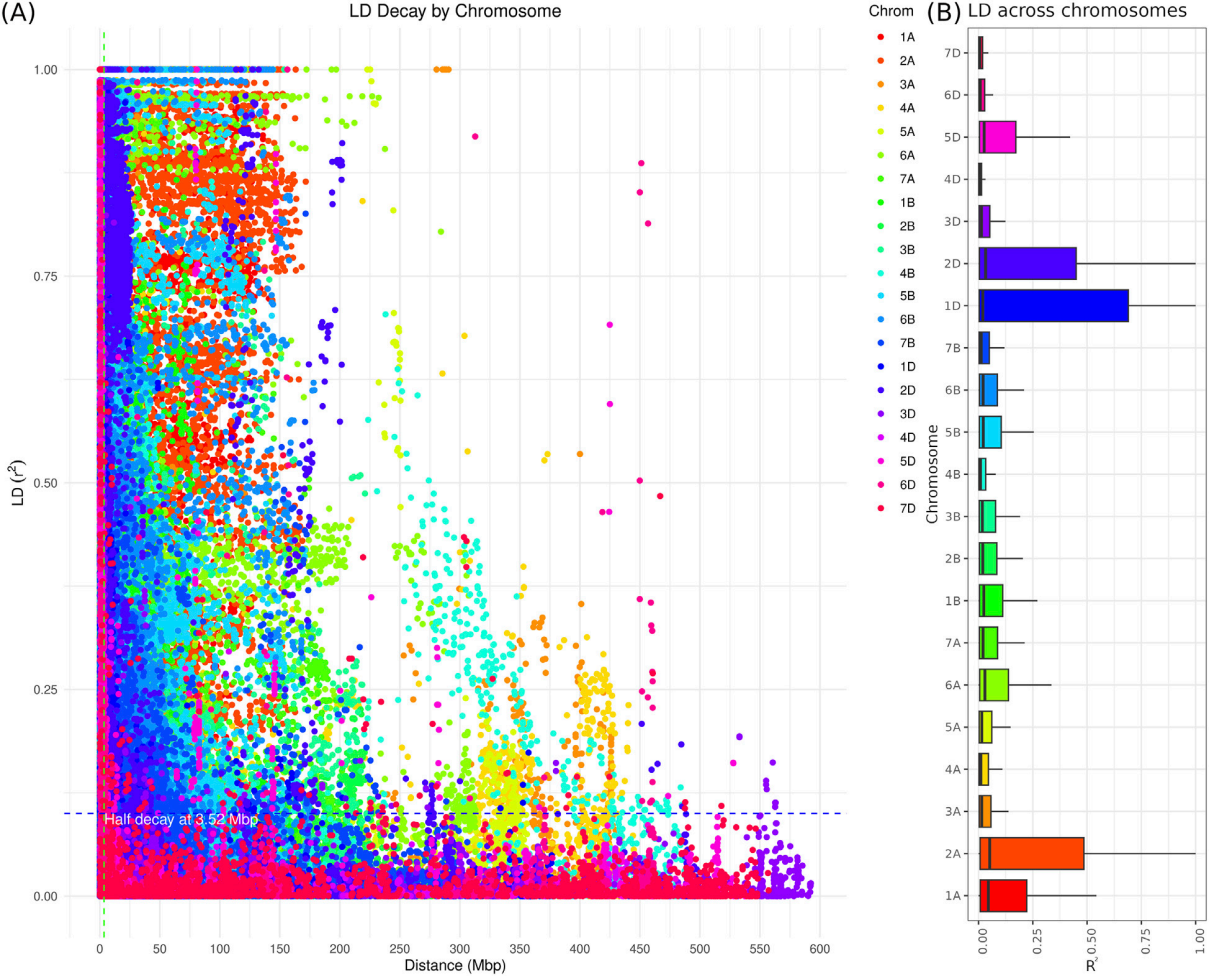
The genomic linkage disequilibrium association across the wheat genome based on 35K Axiom Wheat Genotyping Array data. **(A)** LD decay plot: The X-axis represents the physical distance in base pairs (bp) between SNPs, and the Y-axis represents the LD measured as $R^{2}$ . Red points indicate $r^{2}$ values between SNP pairs, while gray points represent background LD values. The green line marks the threshold for significant LD, and the blue line shows the average $R^{2}$ value across distances. **(B)** Box plot of the LD values across wheat chromosomes.

The linkage disequilibrium (LD) analysis provided using SNP demonstrated how closely SNPs are have a high LD values (Figure 4). This pattern aligns with the expectation that nearby loci are more likely to be in LD. The green line denotes a significance threshold for LD, with values above this line being considered significant, while the blue line provides the average $r^{2}$ value for comparison, which was 35 Kbp in our study. The average $R^{2}$ values across different wheat chromosomes reveal significant variation. Chromosomes 1A–7A and 1B–7B show LD values ranging from very low (0.000) to high (up to 1.000). Notably, chromosome 2A has a higher median LD (0.0516) compared to chromosome 4A (0.0104), indicating generally higher LD on 2A. Conversely, chromosomes 1B–7B have lower median LD values (0.0174–0.0243) (Figure 4). Chromosomes 1D to 7D exhibit a broader range of LD values, with chromosome 1D having a maximum LD of 1.000. Chromosome 5D has a relatively high 3rd quartile (0.1718) among D chromosomes. The D genome generally shows higher median and maximum LD values compared to A and B genomes, suggesting differing recombination rates or selection pressures. Some chromosomes, like 7A and 4B, demonstrate notable variability, with 7A having a high 3rd quartile (0.4852) and 4B a much lower one (0.0329) (Figure 4).

## 4 The genetic structure of the Watkins landrace population reveals three distinct clusters and four ancestral groups

**FIGURE 5:**
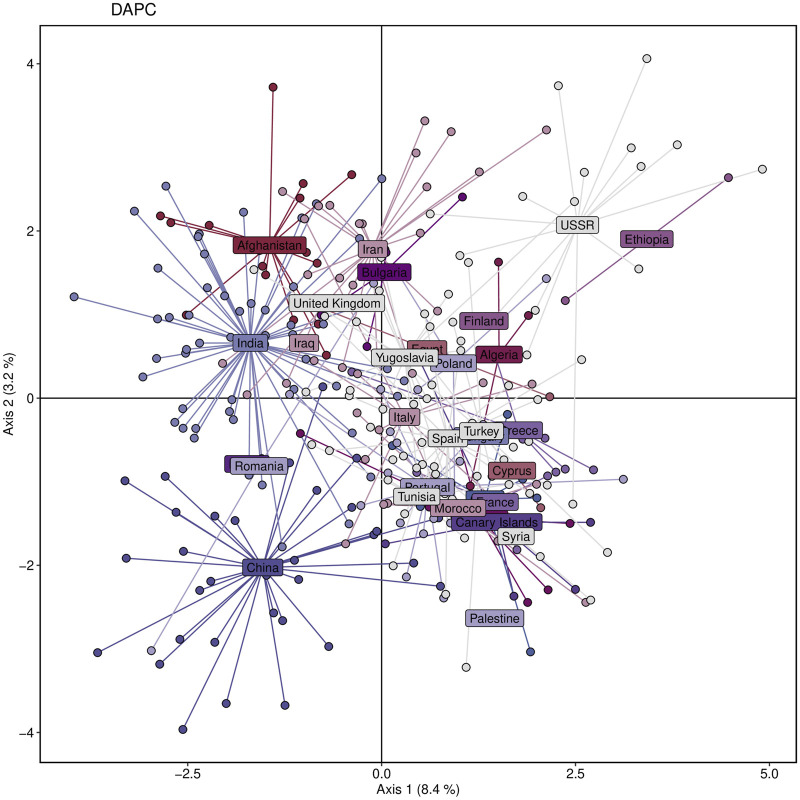
Genetic diversity of the studied wheat population based on Discriminant Analysis of Principal Components (DAPC), showing clustering of individuals according to their sampling countries. Each color represents a different country.

We aimed to investigate the genetic structure and diversity of the Watkins wheat landraces from different countries. To address this, we performed a DAPC analysis based on principal components and discriminant analysis eigenvalues, which grouped the populations into three clusters. Based on the results of the 20 principal components and three discriminant analysis eigenvalues, the DAPC analysis revealed the genetic structure of the entire dataset by grouping populations into three clusters (Figure 5). The study revealed that populations from Asian countries, such as Iran, Afghanistan, India, and Iraq, primarily belonged to a single cluster. On the other hand, populations from Europe, the Middle East, and Africa, including Italy, Spain, France, Palestine, Syria, Egypt, Algeria, and Ethiopia, were predominantly represented in a second cluster. Accessions from China formed a separate cluster distinct from the other groups (Figure 5).

**FIGURE 6:**
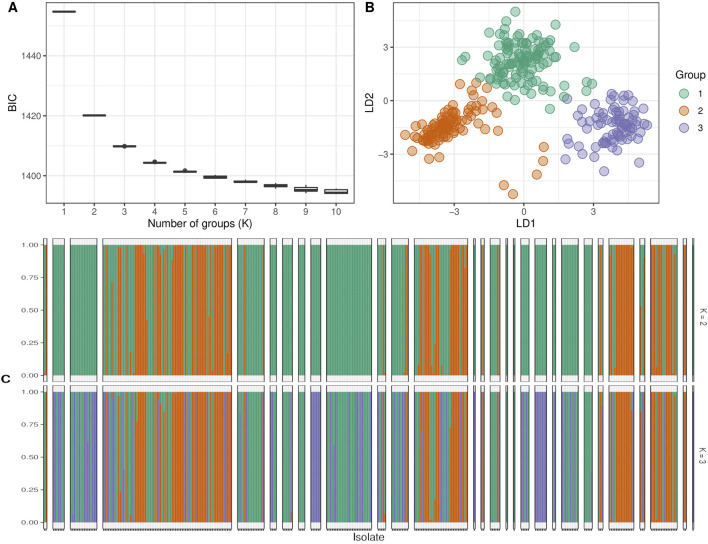
The clustering analysis of the studied wheat landraces. Panel **(A)** shows the expected number of populations for different Bayesian Information Criteria (BIC) values, where the optimal number of populations was predicted to be 3. Panel **(B)** displays the predicted number of genetic clusters based on the DAPC analysis. Panel **(C)** presents a bar plot illustrating the assignment of individual landraces to different groups.

**FIGURE 7:**
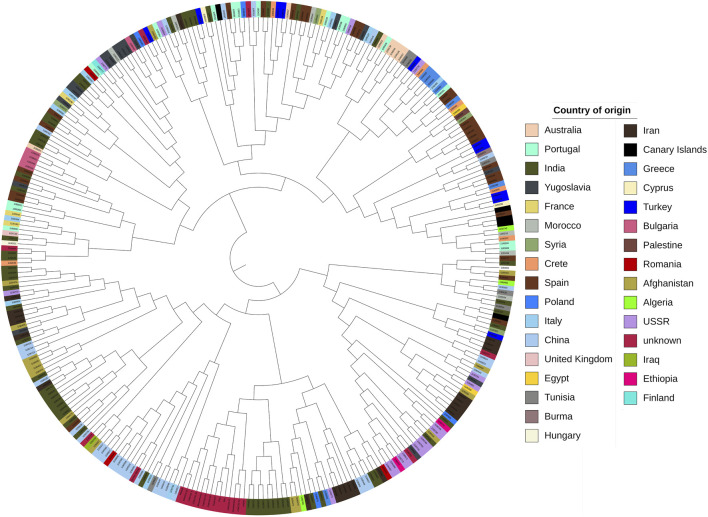
Hierarchical cluster dendrogram of the studied wheat landraces based on SNP genotyping data. The different colors represent the geographical origin of the landraces.

Subsequently, we determined the optimal number of sub-populations using the Bayesian Information Criterion (BIC) method and confirmed it with scatter and bar plots. The analysis of sub-populations using the BIC method showed that the optimal number of sub-populations was three. This conclusion was supported by the lowest value of the BIC curve at three sub-populations (Figure 6A), which was further confirmed by the scatter plot (Figure 6B) and barplot (Figure 6C). The number of three sub-populations agrees with a STRUCTURE analysis conducted on the full Watkins collection (Winfield et al., 2018), which suggests that the chosen panel covers most of the diversity of the full Watkins collection. The barplot analysis revealed that some countries, such as India and China, contain mixed samples from other countries, such as Afghanistan. Therefore, we created a hierarchical cluster dendrogram to investigate gene flow between countries. We identified four distinct ancestral groups from this structural analysis of the population. The clustering algorithm created two primary clusters, supporting the results obtained from the DAPC analysis. The tree also showed evidence of gene flow between countries, which could be due to historical factors such as trade and migration. However, no clear association was observed between genetic clustering and subpopulation (Figure 7).

**FIGURE 8:**
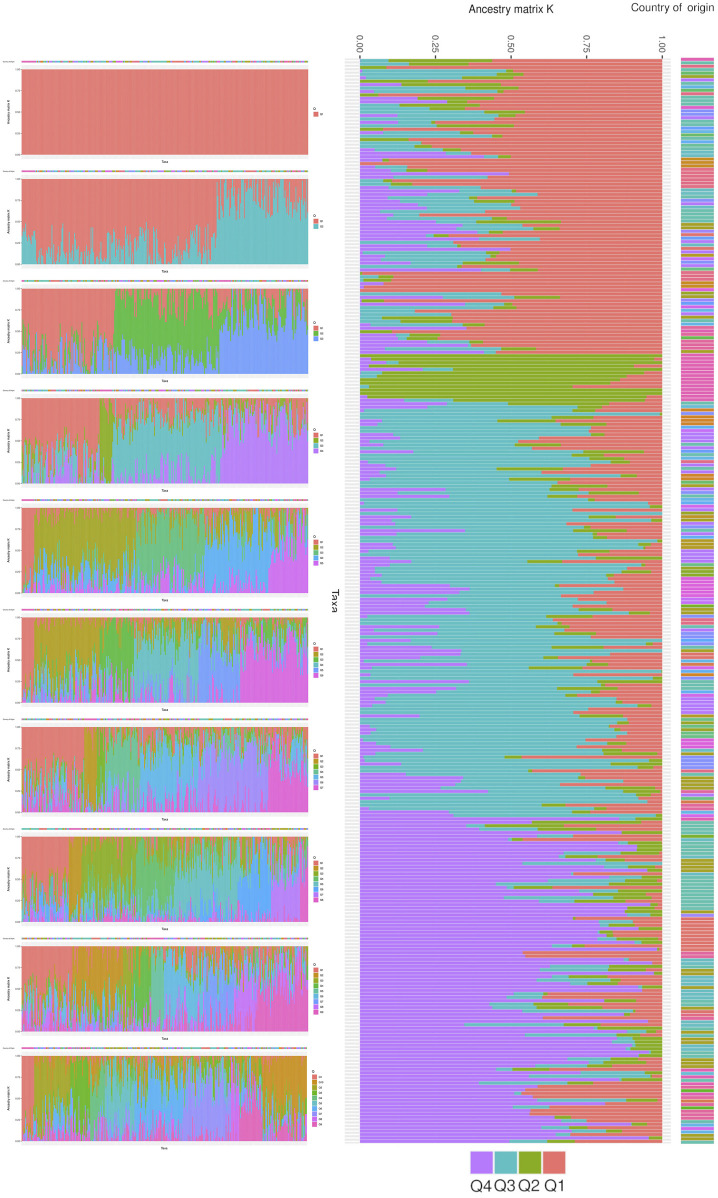
The population structure of studied wheat landraces based on 35K Axiom Wheat Genotyping Array data. The optimal number of ancestral populations was determined using the results of 10 K runs, with K = 4 being the most significant structure for the population. The figure shows the clustering of 316 wheat accessions into four groups (Q1, Q2, Q3, Q4) with different lineages, each representing a specific proportion of the total population.

The core collection of ancestral populations was determined through a structural analysis. This analysis was performed on populations with ancestry values ranging from one to ten (Figure 8). The analysis resulted in four distinct groups from various lineages, as depicted in the optimal ancestry. Group Q1, which accounts for 27% of the population, includes two landraces from Afghanistan, five from Bulgaria, sixteen from India, and eleven from Yugoslavia. Group Q2, the smallest and purest group, represents only 4% of the landraces and contains most of the unknown landraces. Group Q3, the largest group, constitutes 37% of the landraces with representation from China (10 landraces), India (11 landraces), Portugal (12 landraces), Turkey (9 landraces), and Spain (21 landraces). Finally, Group Q4 accounts for 30% of the structural population and includes landraces from Afghanistan (11 landraces), China (17 landraces), India (28 landraces), the former USSR (9 landraces), and five unknown locations.

### 4.1 Genome-wide association analysis

**FIGURE 9:**
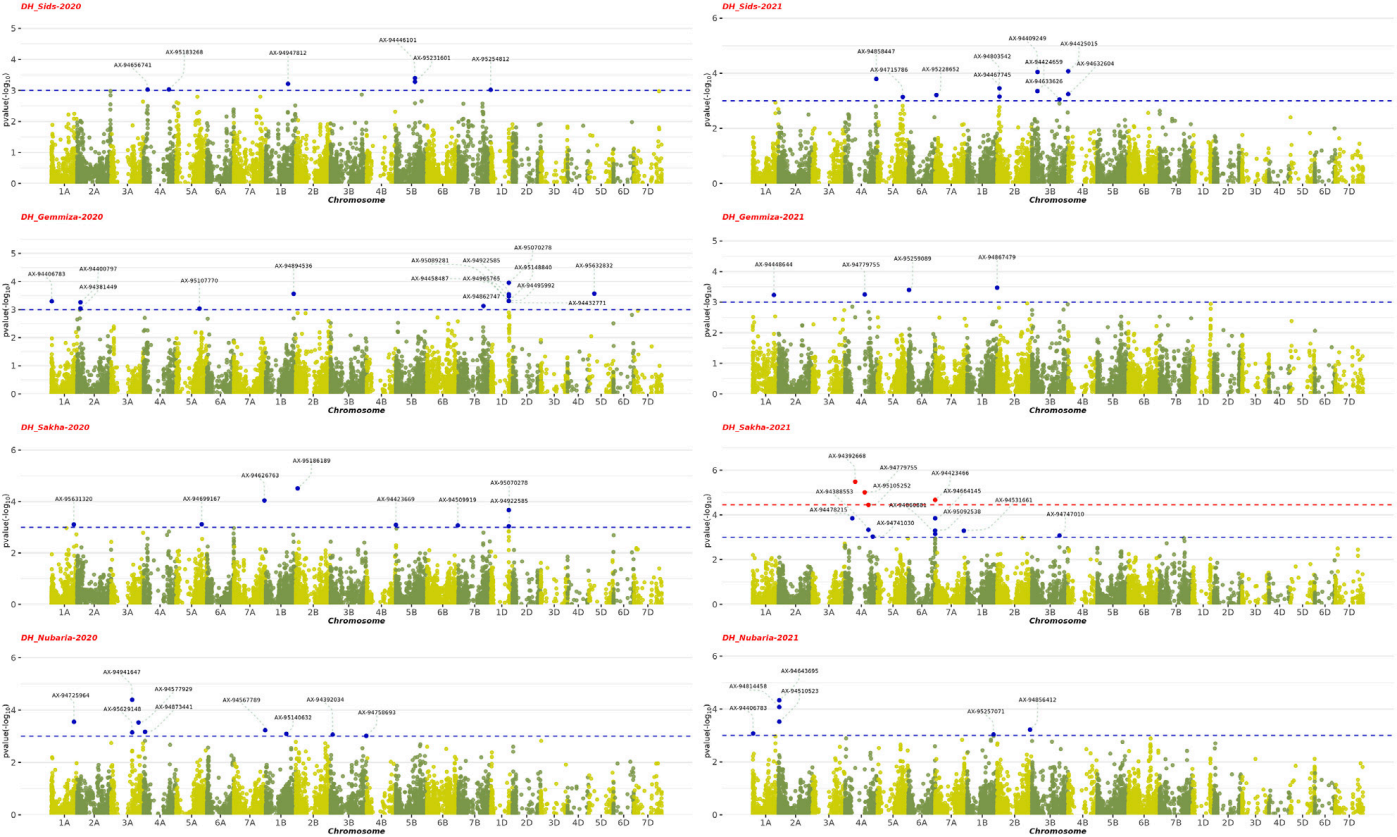
Manhattan plots show the Genome-Wide Association Study (GWAS) results of the days to heading (DH) trait across four environments from 2020 to 2021 and their statistical significance (FDR). Each dot represents a Single Nucleotide Polymorphism (SNP), and its position on the plot represents its chromosomal location. The red dots represent SNPs that are significantly associated with days to heading (DH) trait (FDR ≤ 0.1), while blue dots represent significant SNPs with p-value ≤ 0.001).

**FIGURE 10:**
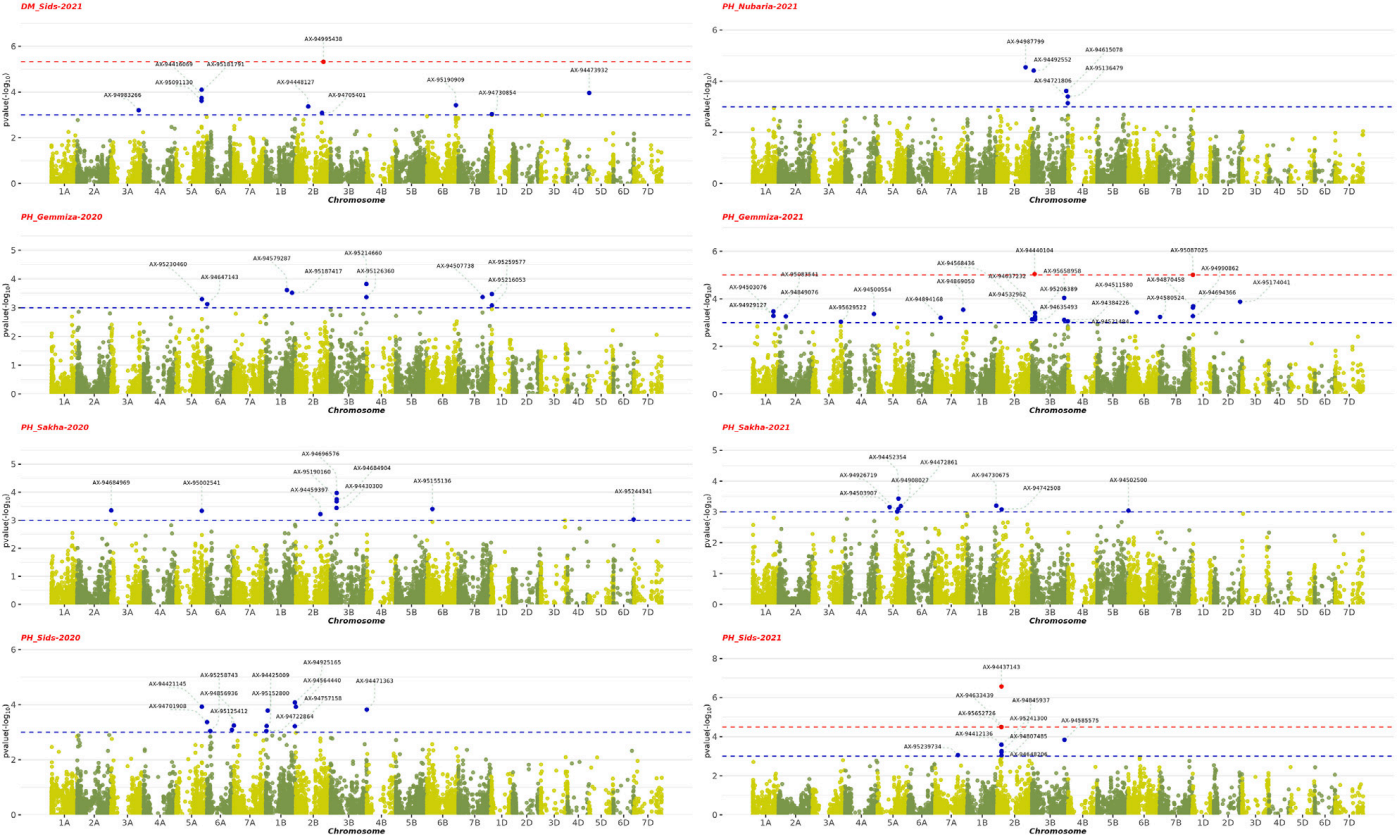
Manhattan plots showing the genome-wide association study (GWAS) results of plant height (PH) and days to maturity (DM) traits across four environments from 2020–2021 to 2021–2022. The x-axis represents the physical position of SNPs on each chromosome, and the y-axis shows the negative logarithm of the adjusted p-values [−log10 (p-value)]. The red dots represent SNPs that are significantly associated with the studied traits (FDR ≤ 0.1), while blue dots represent significant SNPs with p-value ≤ 0.001).

**FIGURE 11:**
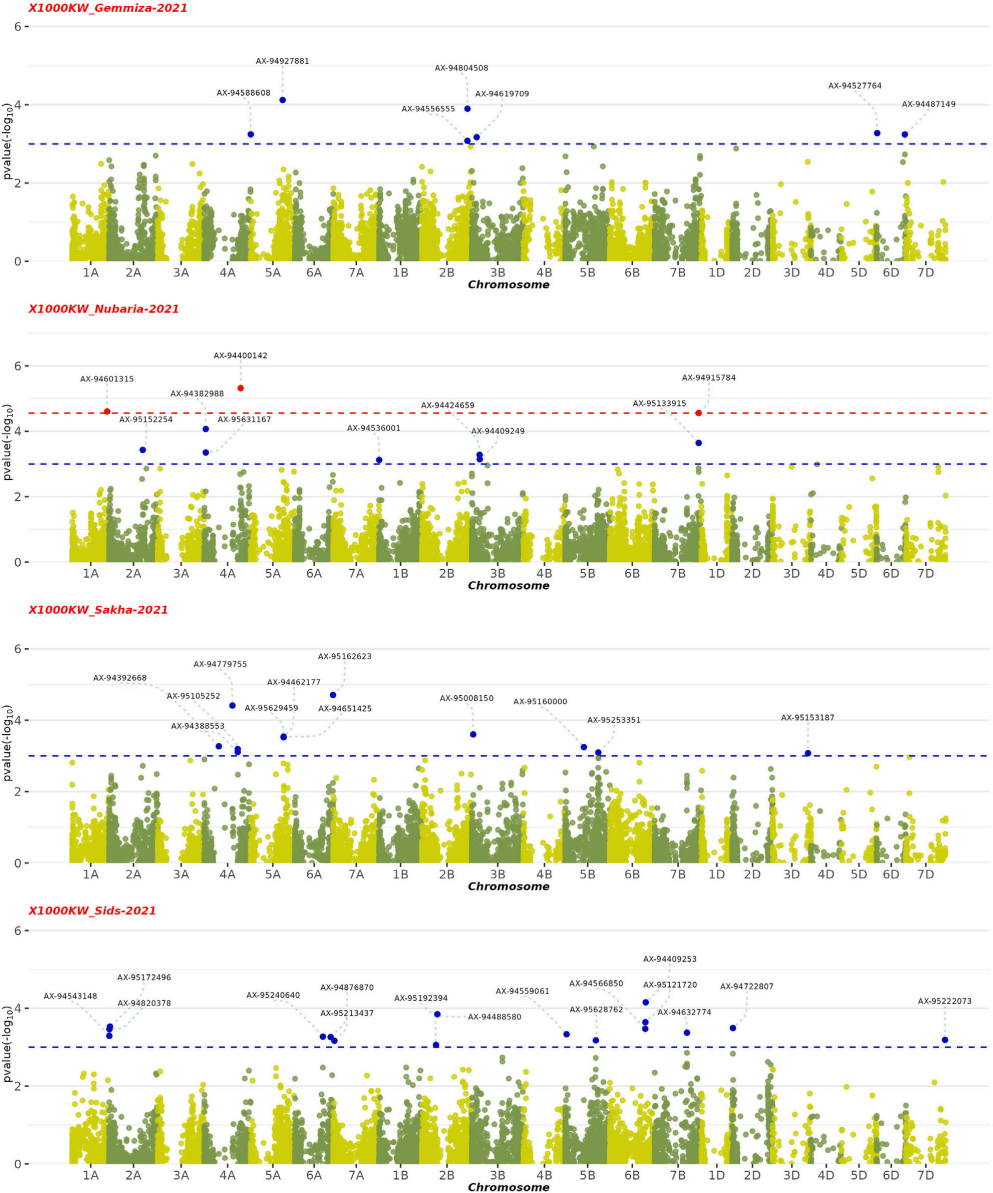
Manhattan plots displaying the genome-wide association study (GWAS) results for the 1,000 kernel weight (1000 KW) trait in wheat across four environments from 2020–2021 to 2021–2022. The horizontal axis shows the physical position of each single nucleotide polymorphism (SNP) across the wheat genome, while the vertical axis represents the –log10 p-value of association for each SNP. The red dots represent SNPs that are significantly associated with the 1,000 KW trait (FDR ≤ 0.1), while blue dots represent significant SNPs with p-value ≤ 0.001).

The GWAS results showed the presence of 202 significant SNPs distributed across all chromosomes, with 3B having the highest frequency (30 SNPs) and 3D and 7D having the lowest (1 SNP) (Supplementary Table S5). Among the four growth indices, plant height was found to be most associated with 77 SNPs, followed by date of heading with 70 SNPs, thousand kernel weight with 44 SNPs, and date of maturity with 10 SNPs (Figures 9–11). The SNP variants presented themselves in several forms of nucleotide changes. The most common change observed was T (thymine) to C (cytosine), with a total of 44 occurrences and an even distribution across all traits. The second most frequent change was C (cytosine) to T (thymine), which was recorded 43 times. The association analysis of thousand kernel weight found significant SNPs on all chromosomes except for chromosomes 3A, 1B, 4B, 1D, 4D, and 5D. The highest number of SNPs affecting this trait was observed on 4A (7 SNPs), followed by 5A (5 SNPs). The trait was also associated with SNPs on chromosomes 2A, 2B, 3B, and 5B (4 SNPs each), chromosomes 6A, 6B, and 7B (3 SNPs each), chromosomes 2A and 6D (2 SNPs each), and chromosomes 1A, 2D, 3D, and 7D (1 SNP each) (Figure 9). All chromosomes except chromosomes 5B, 3D, 4D, 5D, and 7D had SNPs that associated with plant height. The highest number of SNPs associated with this phenotype were found on chromosome 3B, with 20 SNPs, followed by 12 SNPs on chromosome 2B, 8 SNPs on chromosomes 5A and 1B, 6 SNPs on chromosome 1D, 4 SNPs on chromosomes 6A and 7A, 3 SNPs on chromosomes 1A and 6B, 2 SNPs on chromosomes 3A and 7B, and 1 SNP on chromosomes 2A, 4A, 4B, 5B and 2D (Figure 10). All chromosomes except chromosomes 2D, 3D, 4D, 6D, and 7D were found to influence days to heading. Chromosomes 4A and 1D had the highest frequency of SNPs (11 and 10, respectively), while chromosomes 6B and 3D had the lowest (1 SNP each). The remaining SNPs associated with this trait were evenly distributed across the genome (Figure 3). These genotypic variations resulted in phenotypic changes that also influenced plant maturity. One SNP marker associated with this trait was found on chromosomes 3A, 6B, 1D, and 5D, while chromosomes 5A and 2B contained 3 SNPs (Figure 11). We observed only DH, have SNPs shared across different years and stations. Specifically, SNP AX-94406783 was present in the Gemmiza station in 2020 and again in Nubaria in 2021, while SNPs AX-95070278 and AX-94922585 were identified in both Gemmiza in 2020 and Sakha in 2020, highlighting their stability across diverse growing conditions. Additionally, SNP AX-94779755 was detected at Gemmiza in 2021 and Sakha in 2021, further emphasizing its potential relevance to the DH trait across different environments.

### 4.2 Discussion

The evaluated Watkins landraces demonstrated remarkable stability for all tested agronomic traits, including PH, DH, and 1000KW, across diverse growing conditions. Nine specific accessions: BecardKachu, CIMCOG_3, CIMCOG_32, CIMCOG_47, CIMCOG_49, CIMCOG_53, Reedling, Super 152, and Waxwing—consistently performed well across multiple locations. Additionally, six wheat genotypes—BAJ, CIMCOG_56, MISR1, Pfau, Weebill, and Wyalkatchem—showed stability in plant height and days to heading across all trial sites. Several crosses were initiated between Egyptian wheat cultivars and stable accessions. CIMCOG_32 and CIMCOG_47 were selected for the 2023–2024 crossing block. CIMCOG_32 was used as a parent in multiple crosses, including with the Egyptian cultivar Sakha 95 and advanced exotic wheat lines. These efforts underscore the potential of CIMCOG_32 and Watkins landraces in breeding programs to enhance wheat stability and performance across different environments.

Population structure is a statistical technique used to clarify the genetic composition of individuals within a population as well as the ancestry ratio, demonstrating genetic variance among populations (Patterson et al., 2006). It is mainly used for identifying Subpopulations clusters within a larger population, understanding genetic relationships, and depends on PCA analysis to reduce the dimensionality of genetic data and visualizes genetic variation among samples (Patterson et al., 2006). We assessed genetic structure in a collection of 316 global bread wheat genotypes (*Triticum aestivum*) representing mostly landraces. The panel was genotyped with the 35K Axiom(B) Wheat Breeder’s Genotyping Array (Affymetrix product ID 550524). The array contains 35,143 SNPs selected to be informative across a diverse global collection of elite and landrace varieties of hexaploid and tetraploid wheats (Winfield et al., 2018). We filtered the markers based on minor allele frequency and missing data and found that our shortlist of 9,402 SNP markers had a genome distribution consistent with earlier research including a two to five times lower marker density in the D genome (Alipour et al., 2017; Allen et al., 2013). The number of anchored markers is largest in the B genome, followed by the A genome, and lowest in the D genome.

There is significant variation in average $R^{2}$ values across different wheat chromosomes. Chromosomes 1A–7A and 1B–7B show LD values ranging from very low to high, with 1B–7B generally having lower median LD values. Chromosomes 1D to 7D exhibit a broader range of LD values, with higher values observed in the D genome compared to the A and B genomes, indicating differing recombination rates or selection pressures. Notable variability is seen in chromosomes like 7A and 4B. This observed trend is consistent with previous wheat studies. For instance, Mourad et al. (2020) reported that the D genome had the highest significant LD, with an average $R^{2}$ of 0.887, followed by the A and B genomes. Roncallo et al. (2021), found that 13.4% of marker pairs exhibited significant LD, with a high LD ( $r^{2}>0.7$ ) in only 0.94% of comparisons, and an LD decay of 11.8 Mb. Additionally, Aleksandrov et al. (2021), noted that in an old germplasm collection, significant LD ranged from 6% to 27% across different chromosomes, while in a modern collection, it ranged from 10% to 45%. These findings align with our results, which indicate considerable variation in LD across different wheat chromosomes and higher LD values in the D genome compared to the A and B genomes.

A cluster is a group of objects that are closer and more similar to each other than those outside of the group (Grabowski et al., 2018). The DAPC analysis revealed three distinct clusters within the populations, with a total diversity score of 11.6 across both axes. This suggests that while the DAPC could identify relatively low diversity ratios between samples, there is substantial genetic diversity among them. This value helps us understand the extent of variation captured by the analysis. This ratio is relatively low compared to the 60.1% of total variance reported by Fiore et al. (2019) for their study involving twenty-seven durum wheat varieties and one bread wheat Sicilian landrace. This suggests that our observed diversity, as indicated by the sum of 11.6, is lower relative to their findings, potentially indicating less pronounced clustering or different levels of genetic variation in our sample. This value is relatively high compared to the second and third principal components reported by Alemu et al. (2020) in their study of Ethiopian durum wheat (Triticum turgidum ssp. durum). Their study highlighted different patterns of variance, suggesting that our results reflect a higher level of diversity or variability in the principal components analyzed. Afghanistan, Iran, India, China, Iraq, and Burma comprise the first cluster (the majority of the Asian cluster). The second cluster includes most European countries, including Italy, Spain, Portugal, Turkey, and Greece. The third cluster includes African countries such as Egypt, Algeria, and Ethiopia, which are not in the same cluster but are close. The Bayesian information criterion (BIC) is a well-known and commonly used method in statistical model selection. It was applied to approximate a transformation of the Bayesian posterior probability of a candidate model (Neath and Cavanaugh, 2012). To identify the optimum cluster (K)(knee), we displayed BIC in three forms, depending on the DAPC; the first plot was a boxplot, which showed that three and four were significant values for K. The second was a scatter plot that classified the samples into three categories. The third was a bar plot that performed with K = 2 and K = 3 and identified those three as the best group. The clustering findings are consistent with the population structure, which revealed that while K = 4 is the best, K = 2 and K = 3 are also significant. It should be highlighted that the current results of population structure analysis are consistent with our previous findings of the entire set (804 accessions) of hexaploid wheat (Winfield et al., 2018). This consistency reaffirms the reliability of our present findings, indicating a robust and persistent population structure across diverse datasets. Our findings on the clustering of the Watkins landrace populations could provide complementary insights to Pont et al. (2019) identification of selection footprints and evolutionary history in modern wheat. Both studies emphasize the complex genetic landscape of wheat and contribute to a broader understanding of its genetic evolution and diversity.

We conducted a comprehensive GWAS to explore the influence of single nucleotide polymorphism (SNP) markers located on genes controlling four important traits in wheat: days to heading, days of maturity, plant height, and 1,000 kernel weight. The identified SNPs have potential as marker-trait associations (MTAs) to guide breeding programs in the agricultural sector. The analysis of 203 SNP markers yielded 31 genomic regions associated with the studied traits. Notably, three SNPs on chromosome 5A were found to be linked to 1,000 kernel weight, with a physical distance of less than 10 Mb. Additionally, four clusters evenly distributed on chromosomes 3B and 1D were identified as influencing plant height. Thirteen SNPs in clusters on chromosomes 2A, 5B, 6B, and 1D were associated with days to heading. For the trait of maturity date, three SNPs were located on chromosomes 2A, 2B, and 6B. Interestingly, six SNPs were found to be linked to both 1,000 kernel weight and days to heading. These results provide valuable insights for breeders seeking to improve these important traits in wheat and lay the foundation for further functional studies on the identified markers.

To map the detected probes onto the wheat genome and identify candidate genes and encoding protein domains influencing the traits, the GrainGenes database (https://graingenes.org/GG3/) was utilized. Notable associations were observed between specific SNPs and genes controlling the traits. For example, SNP AX-94462177 with a p-value of 0.0002 and 0.4 FDR, associated with 1,000 kernel weight, was detected within the Kinesin-like protein domains (NPK1/TraesCS5A02G317000), known for its role in seed development in rice; these domains play crucial roles in almost all biological processes in plants (Li et al., 2011). SNPs AX-94643695 and AX-94814458 with p-values of 0.00004 and 0.00008, and FDRs of and 0.3, associated with wheat date of heading, were in the ATP-sulfurylase PUA-like gene (TraesCS2D02G031800), indirectly influencing plant development (Xiao et al., 2022; Khan et al., 2007). SNPs AX-94446101 and AX-95231601 with p-values of 0.0004 and 0.0005, and FDRs of 0.904116745, also associated with date of heading, mapped to the Serine/threonine-protein kinase D6PK-like gene (TraesCS5B02G252500) which serves as a lipid domain-dependent regulator of root epidermal planar polarity in Arabidopsis (Stanislas et al., 2015). A cluster of loci (AX-94510523, AX-94425015, and AX-94632604), with p-values of (0.0003, 0.00008, and 0.00057), and FDRs of (0.7,0.3, and 0.6) associated with date of heading was located in the wheat C2H2 ZINC finger transcription factor gene TraesCS4B02G003500 encoding a C2H2-type zinc finger protein, reported as the best candidate gene in the QTL Qhd.2AS controlling wheat growth and development (Li et al., 2023). SNP AX-94983266 with a p-value of 0.0006 and FDR of 0.5, associated with date of maturity, located to the HAUS1 (HAUS Augmin Like Complex Subunit 1/TraesCS3A02G380800) gene. In Arabidopsis, the AUGMIN complex impacts spindle and phragmoplast microtubule arrays during sexual reproduction (Oh et al., 2016). Lastly, SNP AX-94409249 (p-value of 0.0007, and FDR of 0.6), associated with wheat yield and days to maturity, mapped to the RING finger domain gene TraesCS3B02G139600 on chromosome 3B (Supplementary Table S6). In Arabidopsis and tobacco, RING zinc finger genes are involved in seed development and stress resistance (Xu and Li, 2003; Zeba et al., 2009; Han et al., 2022).

## 5 Conclusion

This study revealed a high level of genetic and phenotypic diversity among the evaluated wheat populations. The field trial results demonstrated a high degree of adaptability among the evaluated genotypes, with some accessions displaying particularly favorable phenotypic traits. This highlights the importance of genetic diversity in wheat breeding, especially considering the challenges posed by changing climates and new end-use demands. Through GWAS we identified marker-trait associations for important agronomic traits in wheat, including days to heading, days to maturity, plant height, and 1,000 kernel weight. These associations, along with the suggested genes, provide molecular means to support targeted breeding efforts. Notably, our evaluation of a highly diverse panel of international genotypes under Egyptian climate and agronomic conditions, underscores the potential for utilizing this diversity in developing locally adapted wheat varieties. By capitalizing on these findings, breeders can drive progress towards resilient and high-yielding wheat varieties, ensuring sustainable agriculture and addressing food security challenges in Egypt and beyond.

## Data Availability

The data presented in the study are deposited in the Zenodo repository, accession number https://zenodo.org/records/14035695.
