# Supplementary material for: Assessment of genetic structure and trait associations of Watkins wheat landraces under Egyptian field conditions
Source: Front Genet. 2024 Dec 2;15:1384220. doi: 10.3389/fgene.2024.1384220 (PMC11646717; doi:10.3389/fgene.2024.1384220)
Supplement: Supplementary file 1 [file Table3.DOCX]

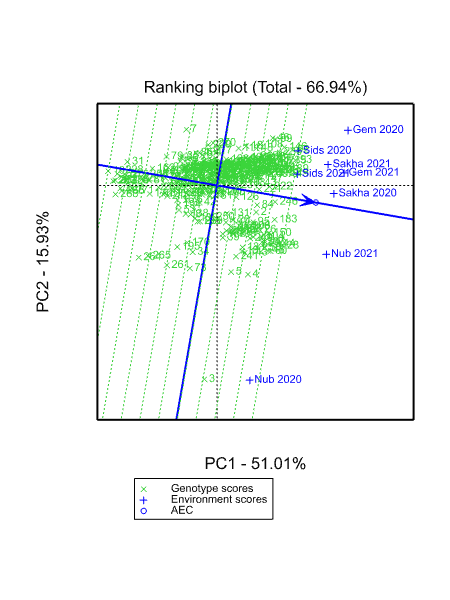


**Supplementary figure 1. The mean vs. stability view of the GGE biplot for the days to heading (DH).**


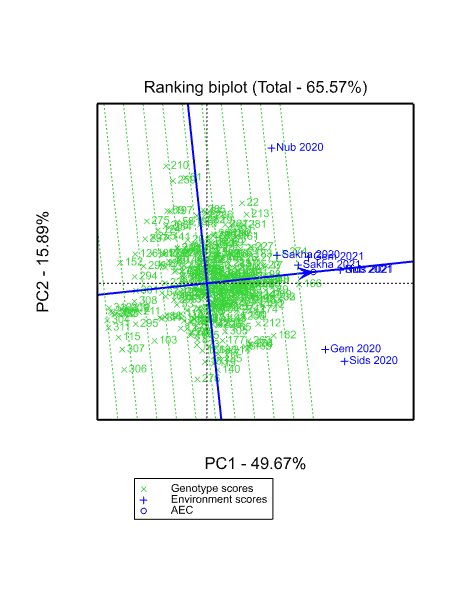


**Supplementary figure 2. The mean vs. stability view of the GGE biplot for plant height.**


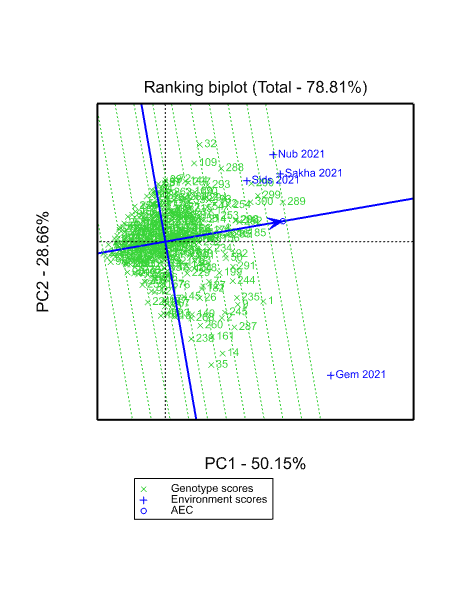


**Supplementary figure 3. The mean vs. stability view of the GGE biplot for 1000 kernel weight.**
